# Highly sensitive detection of lipopolysaccharides using an aptasensor based on hybridization chain reaction

**DOI:** 10.1038/srep29524

**Published:** 2016-07-12

**Authors:** Peiyan Xie, Longjiao Zhu, Xiangli Shao, Kunlun Huang, Jingjing Tian, Wentao Xu

**Affiliations:** 1 Beijing Laboratory for Food Quality and Safety, College of Food Science and Nutritional Engineering, China Agricultural University, Beijing 100083, China; 2 Beijing Advanced Innovation Center for Food Nutrition and Human Health, College of Food Science & Nutritional Engineering, China Agricultural University, Beijing, 100083, China

## Abstract

Lipopolysaccharides (LPS), integral components of the outer membrane of all gram-negative bacteria, are closely associated with foodborne diseases such as fever, diarrhea and hypotension, and thus, the early and sensitive detection of LPS is necessary. In this study, an aptasensor assay based on hybridization chain reaction (HCR) was developed to detect LPS. Briefly, two complementary stable species of biotinylated DNA hairpins coexisted in solution until the introduction of a detection probe triggered a hybridization chain reaction cascade. The DNA conjugates specifically reacted with the LPS, which were captured by the ethanolamine aptamer attached to the reaction well surface. After optimizing the key reaction conditions, such as the reaction time of HCR, the amount of the capture probe and detection probes, the increase in the LPS concentration was readily measured by the optical density value, and a relatively low detection limit (1.73 ng/mL) was obtained, with a linear response range of 1–10^5 ^ng/mL. The approach presented herein introduced the use of an aptasensor for LPS discrimination and HCR for signal amplification, offering a promising option for detecting LPS.

Lipopolysaccharides (LPS), also known as endotoxins, are integral components of the outer membrane of all gram-negative bacteria and consist of the following three distinct regions: O-specific antigen, core polysaccharide and lipid A^1^,^2^,^3^. The core polysaccharide, the stable component of all types of LPS, consists of two or more units of 2-keto-3-deoxyoctonic acid (KDO) linked to two or three L-glycero-D-manno-heptose (Hep), which are only present in bacteria^4^. The hydroxyl group in Hep and KDO is often replaced by ethanolamine^5^. Because LPS is responsible for pyrogenic reaction, septic shock, diarrhea, hypotension and vascular blood clotting, the detection of LPS is essential for medical, pharmacological and food safety^6^.

In the European and Chinese pharmacopoeia, the standard LPS detection method is the Limulus amebocyte lysate (LAL) assay^7^. LAL is based on the clotting reaction between Limulus and LPS, but usually takes several hours, and false positive results can occur because Limulus can react with other LAL-reactive materials, such as β-(1,3)-D-glucan^8^,^9^,^10^. Silver staining is another popular LPS detection method. After silver staining, LPS can be separated by polyacrylamide gel electrophoresis^11^. Silver staining offers an inexpensive detection method using simple equipment and materials. Nonetheless, to visualize the gel-separated LPS, potentially hazardous formaldehyde must still be used, as it is an indispensable silver reductant^12^.

Recently, aptamers have been used in the detection of LPS^13^,^14^. Aptamers, artificial DNA/RNA oligonucleotides selected *in vitro* by SELEX, can fold into well-ordered, three-dimensional molecular architectures in which the ligand becomes an intrinsic part of the nucleic acid structure^15^. Compared to LAL and silver staining, aptamers have the obvious advantages of high affinity, high specificity, chemical stability, strong adaptability and stability in the detection environment. Su *et al.*, reported an aptamer method for LPS detection that combined a gold nanoparticle/PEDOT (polymerized self-assembled monolayer of thiol-functionalized 3,4-ethylenedioxythiophene derivative, PEDOT) platform, which indicates that this method need more requirements for the detection environment^16^.

To increase the sensitivity of target analysis, researchers have introduced signal amplification systems^11^,^17^,^18^. Isothermal amplification technologies that have been used as effective tools to amplify detection signals include rolling circle amplification (RCA)^19^,^20^,^21^ and rapid isothermal detection and amplification (RIDA)^22^,^23^. These methods utilize a primer to extend an oligomer that is attached to the detection target. The extended DNA can then hybridize with multiple oligonucleotides bearing fluorescent probes and thus improve detection limits. However, both technologies rely on isothermal polymerase^24^. Hybridization chain reaction (HCR), which was first proposed by Dirks and Pierce, uses a pair of complementary, kinetically trapped hairpin oligomers to propagate a chain reaction of hybridization events^25^. Compared to RCA and RIDA, the obvious advantage of HCR is that it can amplify at room temperature without enzymes.

Herein, we developed a novel assay that combined HCR for signal amplification with an aptasensor for the detection of LPS using LPS from *E. coli O111:B4* as the model analyte. In detail, biotinylated monomer DNA building blocks were mixed together but did not hybridize on an experimental time scale. Exposure to the detection probe opened the hairpins in the solution and triggered a chain reaction of hybridization events. Afterwards, streptavidin horseradish peroxidase (SA-HRP) was introduced to react with the biotinylated HCR DNA conjugates. Then, the HRP-DNA conjugates were added to 96-well plates where the LPS were captured by the immobilized capture probe and were washed by washing buffer. A visible optical signal only appeared in the presence of the target LPS. Our results showed that HCR for amplification combined with a double-aptamer sandwich method achieved better visual detection of LPS. Compared with traditional assays, this method provides a novel assay for simple, fast, and highly sensitive detection in the field of monitoring LPS.

## Materials and Methods

### Materials and reagents

All chemicals were of analytical reagent grade and used as received. Water was purified by a Millipore Milli-XQ system (Millipore, Bedford, MA, USA). The DNA marker was purchased from Tiangen Biotechnology Co., Ltd (Beijing, China). LPS from *E. coli O111:B4* and LPS from *E. coli O55:B5* were purchased from Sigma-Aldrich Co., Ltd (Shanghai, China). Bovine serum albumin (BSA), streptavidin, tetramethylbenzidine (TMB) and SA-HRP were purchased from Beijing Biyuntian Co., Ltd (Beijing, China). Carboxyl-modified Nunc 96-well plates were purchased from Thermo Fisher Scientific Inc. (Rochester, NY, USA). The human serum samples were purchased from Food safety Technology Co., Ltd (Beijing, China). The nucleic acid sequences (5′-3′) used in this paper^26^,^27^,^28^ were in the Table 1. All oligonucleotides were synthesized by Life Biotechnology Co., Ltd (Beijing, China) and used as received after dissolving in water.

### Apparatus

Visual measurements were carried out on a portable spectrophotometer (NS810, Shenzhen 3nh Technology Co., Ltd). The electrophoresis system consisted of a DYCP-31E vertical electrophoresis tank (Beijing Liuyi Instrument Plant, China) and a steady voltage electrophoresis power supply (PowerPacTM HV, Bio-Rad, USA). Images were recorded by the Gel Doc UV system (Bio-Rad, USA).

### Gel electrophoresis

The target LPS was incubated with 200 nM aptamer consisting of 500 nM H1 and 500 nM H2 in 20 μL of reaction buffer (2.5 mM NaH_2_PO_4_, 8 mM Na_2_HPO_4_, 0.15 M NaCl, 2 mM MgCl_2_, pH 7.4) for 1 h at 37 °C. The agarose gel electrophoresis was run at 130 V for 30 min and then photographed with a digital camera.

### Immobilization of the capture probe

First, 100 μL streptavidin (2 μg/L) in coating buffer (38 mM NaHCO_3_, 15 mM Na_2_CO_3_, pH 9.6) was added to the wells and incubated for 1 h at 37 °C. Then, the wells were washed three times with 150 μL of wash buffer (0.15 M NaCl, 10 mM K_2_HPO_4_, 1 mM NaH_2_PO_4_, 0.05% Tween 20, pH 8) to remove free streptavidin. Next, the samples were blocked with 100 μL of 10% BSA per well at 37 °C for 1 h to minimize non-specific adsorption, and 100 μL biotinylated ethanolamine aptamer solution (100 nM) was added to wells and incubated at 37 °C for 1 h.

### Extension of oligonucleotide chains by HCR

The aptamer and the hairpin oligonucleotides were heated to 95 °C for 2 min and then cooled to room temperature for 1 h before use. A 200 nM LPS aptamer was mixed with 500 nM H1 and 500 nM H2 in 5 mL of reaction buffer (8 mM Na_2_HPO_4_, 2.5 mM NaH_2_PO_4_, 0.15 M NaCl, 2 mM MgCl_2_, pH 7.4) and incubated for 37 °C for 1 h. Afterwards, 5 μL SA-HRP was added to the buffer and shaken at 37 °C for 1 h.

### Peroxidase-Linked Microplate Procedure

After immobilization of the capture probe, 100 μL LPS was added to the wells and reacted at 37 °C for 1 h. The wells were then washed with wash buffer three times to remove free LPS. Next, 50 μL of the prepared HRP-DNA conjugates and 50 μL reaction buffers were added to the wells and incubated for 1 h at 37 °C. The resultant HRP conjugates were washed three times with 150 μL of wash buffer, and then, 100 μL TMB peroxidase substrate was added to the wells to trigger the chromogenic reaction. Add 50 μL 2 M H_2_SO_4_ to the wells after 15 minute for terminating the reaction.

## Results and Discussion

### The principle of the HCR aptasensor

**Figure 1:**
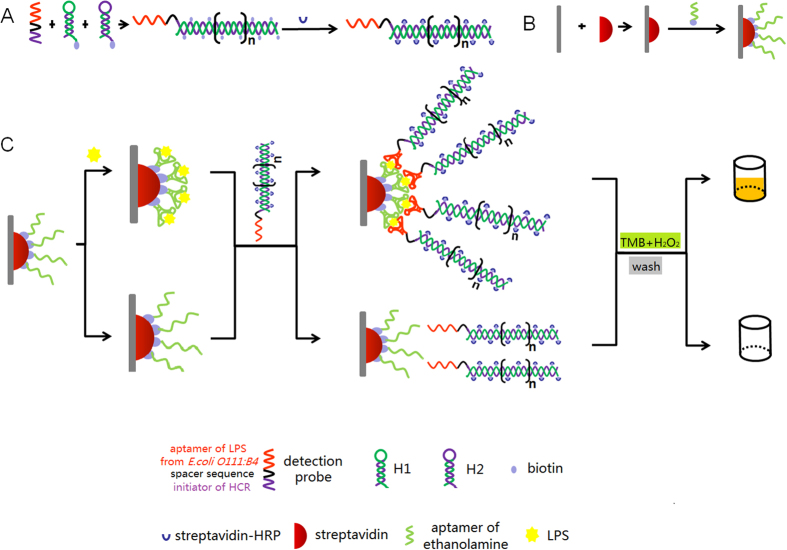
Schematic representation of HCR-based aptasensor for the sensitive detection of LPS.

Non-enzymatically amplified visual measurements based on the aptasensor were used to identify and detect LPS at low concentrations. Scheme 1 illustrates the reaction scheme for the amplified detection of LPS from *E. coli O111:B4*. As shown in Fig. 1, the experiments can be divided into the following three parts: the immobilization of the capture probe, the HCR of the detection aptamer and peroxidase-linked microplate procedure. The first two reactions were carried out at the same time.

To immobilize the capture probe, the streptavidin was incubated in the wells. The biotinylated ethanolamine aptamer was bound in the well by the combination of streptavidin and biotin. In the HCR of the detection aptamer, the two hairpins could not open and hybridize with each other at 37 °C. The introduction of an detection probe strand triggered a chain reaction of alternating kinetic escapes by the two hairpin species into a nicked double helix. In this process, the amplification of the initiator recognition event continued until the supply of H1 or H2 was exhausted. The driving force for this reaction was the formation of polymers triggered by the aptamer and the alternating hybridization between the two hairpins. Because the two fuels were modified with biotin, we used SA-HRP to combine the HCR conjugates to achieve better visual detection. To maximize the hybridization efficiency, HCR was conducted in the solution instead of on a solid surface. Here, we achieved HRP-DNA conjugates as the detection aptamer. When the LPS from *E. coli O111:B4* appeared, the ethanolamine aptamer captured them in the wells. After incubating with HRP-DNA conjugates and washing three times to remove the free LPS, we introduced TMB and H_2_O_2_, which can be reacted under HRP catalysis. The OD values were detected by a portable spectrophotometer.

### Optimization of streptavidin and capture probe

**Figure 2:**
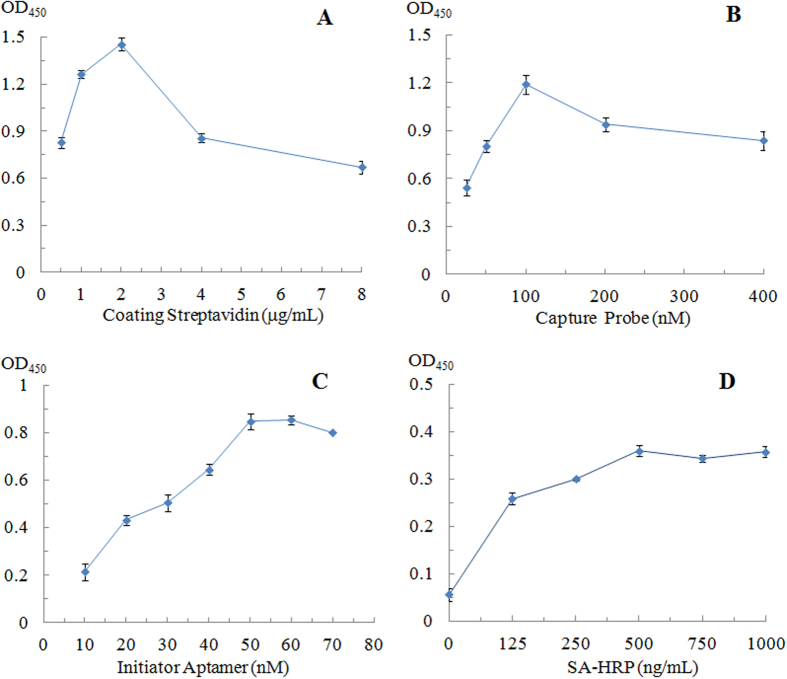
(**A**) The effect of the streptavidin coating. Experimental conditions: the capture probe, LPS, the detection probe, H1/H2 and SA-HRP were 100 nM, 10 μg/mL, 500 nM/500 nM and 500 ng/mL, respectively. (**B**) The effect of the capture probe: the streptavidin coating, LPS, the detection probe, H1/H2 and SA-HRP were 2 μg/mL, 10 μg/mL, 100 nM, 500 nM/500 nM and 500 ng/mL, respectively. (**C**) The effects of detection probe sequence. Experimental conditions: the streptavidin coating, the capture probe, LPS and SA-HRP were 2 μg/mL, 100 nM, 5 μg/mL, and 500 ng/mL, respectively. (**D**) The effects of SA-HRP. Experimental conditions: the streptavidin coating, the capture probe, LPS, the detection probe and H1/H2 were 2 μg/mL, 100 nM, 0.5 μg/mL, 50 nM, and 250 nM/250 nM.

The amount of streptavidin coating the wells plays an important role in the successful immobilization of the capture probe. According to Fig. 2A, the optical density value increased until the streptavidin coating was 2 μg/mL and then decreased as the streptavidin coating increased further. The decrease in the optical density value could be attributed to the saturation of the streptavidin coating on the wells^29^,^30^. Excess streptavidin coating would cause steric hindrance, reducing the binding with biotinylated capture probe.

A range of quantities of ethanolamine aptamer were investigated as shown in Fig. 2B. The optical density value was initially observed to increase with the increasing concentration of the capture probe, reaching a maximum at 100 nM. Further increase in the concentration of the capture probe to 400 nM caused a slight decrease in the optical density value. Thus, 100 nM of the capture probe was selected for subsequent experiments.

### Optimization of HCR

To confirm the hybridization chain reaction, native gel electrophoresis was performed revealing a distribution of polymer lengths. Fig. S1 demonstrates that the hairpins could only polymerize in the presence of the detection probe, and the target LPS did not affect the hybridization of three strands.

The effects of the amount of the detection probe sequence, the concentration of SA-HRP, the HCR reaction time and the reaction temperature were subsequently examined and optimized.

The optical density value increased as the amount of detection probe was increased from 10–50 nM and then decreased slightly as the amount was further increased (Fig. 2C). Thus, 50 nM of the detection probe was selected for subsequent experiments. The optical density value also increased with the concentration of SA-HRP (125–500 ng/mL) and then remained stable with further increases in the SA-HRP concentration (Fig. 2D). Therefore, 500 ng/mL SA-HRP was selected for use in further studies.

**Figure 3:**
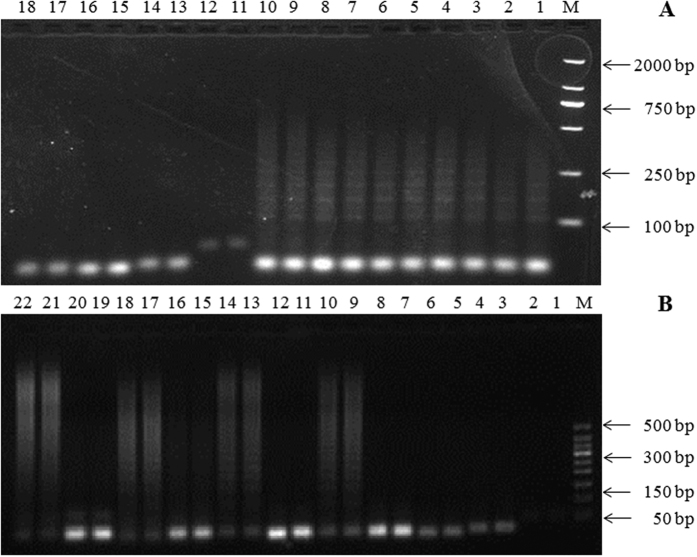
(**A**) The effects of reaction time. Experimental conditions: (1–10) the detection probe andH1/H2 were 50 nM and 250 nM/250 nM, respectively. (1, 2) reaction time was 0.5 h, (3, 4) reaction time was 1 h, (5, 6) reaction time was 2 h, (7, 8) reaction time was 3 h, (9, 10) reaction time was 4 h; (11, 12) 50 nM of the detection probe; (13, 14) 250 nM of H1; (15, 16) 250 nM of H2; (17, 18) 250 nM of H1 and 250 nM of H2, reaction time was 4 h. (**B**) The effects of reaction temperature. Experimental conditions: (1, 2) 50 nM of the detection probe; (3, 4) 250 nM of H1; (5, 6) 250 nM of H2; (7, 8, 11, 12, 15, 16, 19, 20) 250 nM of H1 and 250 nM of H2; (9, 10, 13, 14, 17, 18, 21, 22) the detection probe and H1/H2, were 50 nM, 250 nM/250 nM. (7–10) reaction temperature was 37 °C, (11–14) reaction temperature was 25 °C, (15–18) reaction temperature was 15 °C, (19–22) reaction temperature was 10 °C.

The time spent is an important indicator to evaluate the merits of aptasensors. We could clearly see that for the HCR polymerization, the number of DNA conjunctions increased from 0.5 h to 1 h and then remained almost constant (Fig. 3A). It means until the supply of H1 or H2 was exhausted after 1 h, the amplification of the initiator recognition could complete for 1 h. In order to improve detection efficiency of the aptasensor, we chose 1 h as the HCR. polymerization time for detection. The reaction temperature was critical for detection. We selected 10 °C, 15 °C, 25 °C, and 37 °C as the reaction temperatures, and HCR was triggered at all of these temperatures (Fig. 3B). However, slight diffusion strips appeared in the electrophoresis’s 19–20 rows. There were DNA duplexes whose molecular weight were >150 bp even >500 bp. And DNA duplexes were same with the product of HCR. So we inferred that H1 and H2 could be triggered without the initiator at 15 °C, and this temperature should not be used as the reaction temperature.

### Amplification effect of HCR

**Figure 4:**
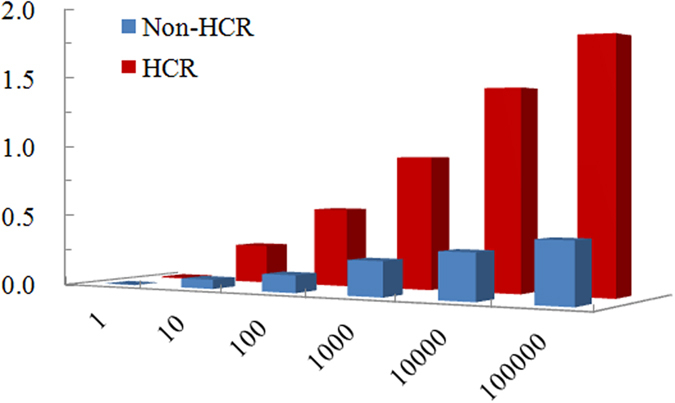
The optical density value vs. different detection methods. Experimental conditions of HCR: capture probe, detection probe, H1/H2 and SA-HRP were 100 nM, 50 nM, 250 nM/250 nM and 500 ng/mL, respectively; Experimental conditions of Non-HCR: capture probe, detection probe and SA-HRP were 100 nM, 50 nM, and 500 ng/mL, respectively; the reaction temperature was 37 °C. The detection procedure was carried out as described in the Experimental section.

To prove the amplification effect of HCR, we compared the HCR aptasensor and a non-HCR aptasensor, which employed a biotinylated aptamer of the LPS from *E. coli O111:B4*. As shown in Fig. 4, the optical density value of the HCR-based system was obviously higher than that of the non-HCR system, and the sensitivity was noticeably improved, clearly indicating that the optical density value incorporated in the HCR process was at least 4-fold greater than that in the non-HCR method.

### Assay performance

**Figure 5:**
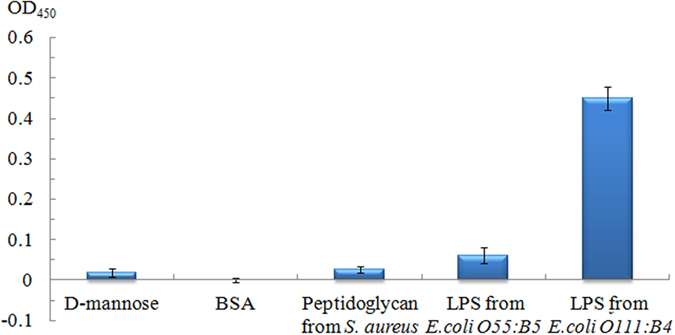
Assay specificity. Experimental conditions: capture probe, detection probe, H1/H2 and SA-QD were 100 nM, 50 nM, 250 nM/250 nM and 500 ng/mL, respectively; and the reaction temperature was 37 °C. The detection procedure was carried out as described in the Experimental section.

Sensitivity and specificity are two important parameters for a successful LPS assay system. In this study, the sensitivity was primarily dependent on the formation of polymerizing hairpins. The detection specificity was basically determined by the function of the capture probe and detection probe. To evaluate the detection specificity of the aptasensor, D-mannose, BSA, peptidoglycan from *Staphylococcus aureus* and LPS from *E. coli O55:B5* were chosen as the non-specific species and investigated. As shown in Fig. 5, high optical density values were only obtained in the presence of LPS from *E. coli O111:B4*. The optical density values were weak in the presence of D-mannose, BSA, peptidoglycan from *Staphylococcus aureus* or LPS from *E. coli O55:B5* at 500 ng/mL and were comparable to the background signal. These data demonstrated that the optical density value was specifically high for the aptamer/target binding, which indicated that the aptasensor we proposed here exhibited good performance for discriminating LPS from *E. coli O111:B4* from other interfering bindings.

### Quantification of LPS

**Figure 6:**
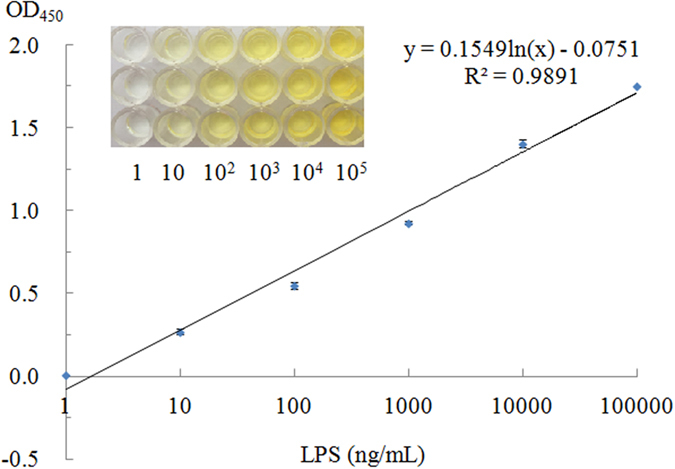
Quantification of LPS. Experimental conditions: the streptavidin coating, the capture probe, the detection probe, H1/H2 and SA-HRP were 2 μg/mL, 100 nM, 50 nM, 250 nM/250 nM and 500 ng/mL, respectively.

Examination of the relationship between optical density value and the amount of LPS revealed a linear relationship in the concentration range of 1–10^5^ ng/mL (Fig. 6), represented by y = 0.1549ln(x) − 0.0751 (R^2^ = 0.9891). The LOD is 1.73 ng/mL, calculated by the standard formulae, as mentioned below and further specified in the literature^31^,^32^,^33^. Each data point was the average of three individual measurements.


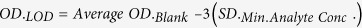


where *OD.*_*LOD*_ is the optical density corresponding to LOD, respectively; *OD.*_*Blank*_ is the optical density of the blank; and SD_.Min Analyte Conc_. is the standard deviations of the minimum analyte concentration, respectively.

### Assay of LPS in drinking water and human serum samples

To evaluate the detection application of this colorimetric method, we carried out a recovery test of LPS in drinking water and human serum. Different concentrations of LPS were added into drinking water and 5-fold diluted human serum samples. The recoveries of LPS detected in the drinking water samples ranged from 97.00% to 103.83% (Table S1). The recoveries of LPS detected in the serum samples ranged from 98.23% to 102.00% (Table S2). These results were satisfactory for quantitative assays performed in biological samples.

## Conclusions

In this paper, a novel HCR-based aptasensor was developed for the highly sensitive detection of LPS. In our design, two kinds of aptamers were introduced which could bind the LPS with high affinity and high specificity. HCR was employed as the amplification tool. A quantitative analysis of target LPS was then carried out by measuring the optical density value. The relatively low detection limit was 1.73 ng/mL, with a linear response range of 1–10^5^ ng/mL. Compared with other methods of LPS detection^34^, our aptasensor based on HCR provides a visual and method with the same detection limitation for the highly sensitive detection of target LPS. In practical applications, the detection probe and the 96 wells which were coated by the capture probe can be prepared in advance, so they can detect the target LPS in less time. This novel technique is isothermal and can easily be adopted for use as an aptasensor for molecular detection.

## Additional Information

**How to cite this article**: Xie, P. *et al.* Highly sensitive detection of lipopolysaccharides using an aptasensor based on hybridization chain reaction. *Sci. Rep.* **6**, 29524; doi: 10.1038/srep29524 (2016).

## Supplementary Material

Supplementary Information

## Figures and Tables

**Table 1:**
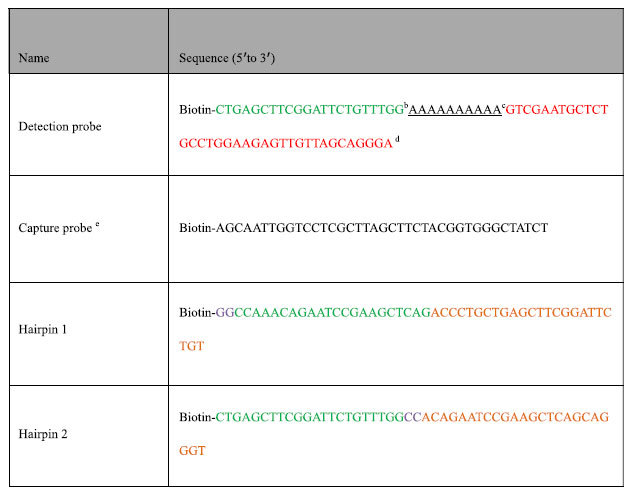
DNA sequences used in this study^a^.

^a^Complementary sequences have the same colors. ^b^Initiator of HCR. ^c^Spacer sequence^26^. ^d^Aptamer of *E. coli O111:B4* LPS^27^. ^e^Aptamer of ethanolamine^28^.
