# Supplementary material for: Highly sensitive detection of lipopolysaccharides using an aptasensor based on hybridization chain reaction
Source: Sci Rep. 2016 Jul 12;6:29524. doi: 10.1038/srep29524 (PMC4941573; doi:10.1038/srep29524)
Supplement: Supplementary Information [file srep29524-s1.doc]

# Supporting information

# Ultrasensitive detection of lipopolysaccharides using an aptasensor based on hybridization chain reaction

Peiyan Xie^2^, Longjiao Zhu^1^, Xiangli Shao^2^, Kunlun Huang^2^, Jingjing Tian^2^, Wentao Xu^1, 2*^

^1^Beijing Advanced Innovation Center for Food Nutrition and Human Health, College of Food Science & Nutritional Engineering, China Agricultural University, Beijing, 100083, China

^2^Laboratory of Food Safety, College of Food Science and Nutritional Engineering, China Agricultural University, Beijing 100083, China

*To whom correspondence should be addressed: Wentao Xu., Tel/Fax: +86 010 62736479., Email: [xuwentao@cau.edu.cn](mailto:xuwentao@cau.edu.cn)


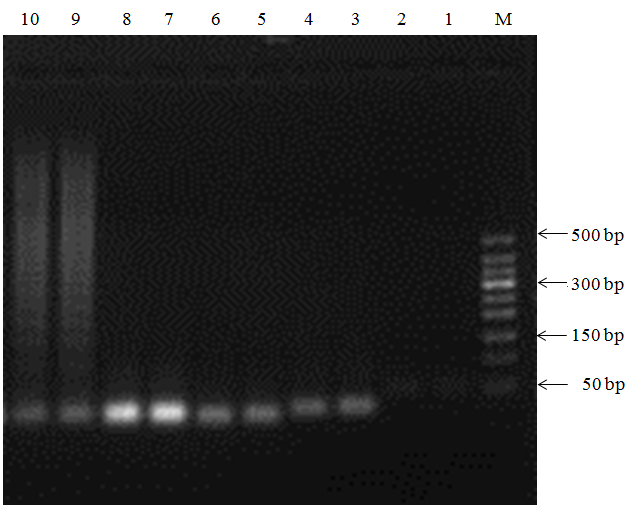


Fig. S1 The confirmation of HCR. (1,2) 50 nM of the initiator aptamer., (3,4) 250 nM of H1., (5,6)

250 nM of H2., (7,8) 250 nM of H1 and 250 nM of H2., (9,10) 50 nM of the initiator aptamer,

250 nM of H1 and 250 nM of H2.

Table S1. Determination of LPS added in drinking water with this proposed method.

| Sample number | Added (μg/mL) | Found (μg/mL) | Recovery (%) |
| --- | --- | --- | --- |
| 1 | 0. 5 | 0.49 | 98.30 |
| 2 | 5 | 5.12 | 102.35 |
| 3 | 10 | 9.95 | 99.45 |
| 4 | 50 | 49.70 | 99.40 |

Table S2. Determination of LPS added in 5-fold diluted normal human serum with this proposed method.

| Sample number | Added (μg/mL) | Found (μg/mL) | Recovery (%) |
| --- | --- | --- | --- |
| 1 | 0.5 | 0.51 | 101.50 |
| 2 | 5 | 5.01 | 100.15 |
| 3 | 10 | 9.68 | 96.75 |
| 4 | 50 | 52.16 | 104.31 |
